# Bumblebee nest departures under low light conditions at sunrise and sunset

**DOI:** 10.1098/rsbl.2023.0518

**Published:** 2024-04-10

**Authors:** Katherine E. Chapman, Michael T. Smith, Kevin J. Gaston, Natalie Hempel de Ibarra

**Affiliations:** ^1^ Centre for Research in Animal Behaviour, Psychology, Faculty of Health and Life Sciences, University of Exeter, Exeter, UK; ^2^ Department of Computer Science, University of Sheffield, Sheffield, UK; ^3^ Environment and Sustainability Institute, University of Exeter, Penryn, Cornwall, UK

**Keywords:** insects, foraging, diurnal, vision, decision-making, behaviour

## Abstract

Only a few diurnal animals, such as bumblebees, extend their activity into the time around sunrise and sunset when illumination levels are low. Low light impairs viewing conditions and increases sensory costs, but whether diurnal insects use low light as a cue to make behavioural decisions is uncertain. To investigate how they decide to initiate foraging at these times of day, we observed bumblebee nest-departure behaviours inside a flight net, under naturally changing light conditions. In brighter light bees did not attempt to return to the nest and departed with minimal delay, as expected. In low light the probability of non-departures increased, as a small number of bees attempted to return after spending time on the departure platform. Additionally, in lower illumination bees spent more time on the platform before flying away, up to 68 s. Our results suggest that bees may assess light conditions once outside the colony to inform the decision to depart. These findings give novel insights into how behavioural decisions are made at the start and the end of a foraging day in diurnal animals when the limits of their vision impose additional costs on foraging efficiency.

## 1. Introduction

Around sunrise and sunset, diurnal animals that are active must contend with low levels of illumination and increasing or declining light intensity. Although these changes occur on a daily basis, little is known about how diurnal insects use this variable visual environment, and whether assessment of the visual conditions mediates the initiation or cessation of their regular behavioural activities. Bees are one such group of insects active during these times. Like most insects, they guide their flight visually with apposition eyes that are typically adapted for vision under bright light [1]. Activity in low light is limited by the sensitivity and capacity of their eyes and visual system [2,3]. While natural dim-light behaviour has previously been described in solitary bees [4–6] and honeybees [2], until recently there was no more than qualitative evidence of bumblebee activity in such conditions. Bumblebees have been observed foraging in the early morning and late evening [7–9], and our recent work has determined that buff-tailed bumblebees *Bombus terrestris* are facultative evening foragers [10]. Individuals which take the opportunity to forage early in the morning or late in the evening may benefit from reduced competition [11,12], access to certain resources [13,14], or extension of their foraging day, but may experience costs of choosing to forage, such as higher likelihood of being unable to navigate [4,15] and reduced pollen acquisition [6,10]. It is therefore of interest to study bumblebees as a model for insects making behavioural decisions under these light conditions.

The initiation of a foraging bout begins within the dark colony-space for cavity-nesting bees. Circadian rhythms set the start of the forager bees' daily activity cycle and play an important role. Previous research conducted in temperate and sub-polar regions has found that forager bumblebees tend to follow a 12 h activity cycle, entrained by natural light fluctuations [16–19]. Within-colony factors, including colony state, may also influence the onset of activity and emergence from the nest [20]. However, it is likely that pre-emergence factors alone are insufficient to ensure that responses are adaptive under fluctuating environmental conditions, thus would not be the sole determinant for a forager's decisions made upon departure from the nest. For example, there is a variable delay within and between individuals from first light to departure in the morning [9]. This suggests that a decision is made to move from within the colony to emerge at the entranceway, where it is then possible for the individual to assess external environmental conditions and choose whether to depart immediately or with a delay, or even return to the nest. It is not known, however, what happens as a forager emerges into the illuminated outer environment and whether decision-making differs between smaller- and larger-sized bumblebee foragers [21,22].

A larger body size correlates with larger eyes [23] and greater visual sensitivity [3], therefore the size of a forager determines its experience of lower light conditions [24]. Alongside other factors relating to size including temperature regulation [25] and memory [22], differing visual sensitivities may result in foragers making different decisions in similar light conditions. Furthermore, size contributes to the initiation of activity at sunrise and start of the day. Larger bees tend to emerge earlier and in lower light conditions, and more experienced small-sized bees utilize these periods [9]. By contrast, at sunset, foragers of all sizes are active in low-light conditions [10], presumably all with accrued experience from the day.

In the present study, we use bumblebees to explore whether diurnal insects may assess light conditions in the uncertain light environment at the start and end of the day to guide behavioural decisions. We aimed to determine whether buff-tailed bumblebee *Bombus terrestris* foragers make a final decision to depart the colony at the nest entranceway when exposed to ambient light, using light intensity as a measure of light conditions. We observed the behaviours of bees emerging onto a platform under natural illumination changes during sunrise and sunset. We analysed the most commonly observed behaviours: the likelihood of the bee attempting to return to the nest; latency to depart from the platform; and walking speed. We additionally analysed duration of inactivity on the platform (see electronic supplementary materials). We expected to see changes in behaviour in low light, near their visual sensitivity threshold [26]. We predicted that if light is assessed upon emergence, bees would attempt to return to the nest under lower light conditions, with a higher probability of returns at sunset and with decreasing body size. Visual constraints could mean that smaller, but not larger, bees may show more delayed departures when in lower and declining light conditions. We predicted that if bees walked on the platform, we would see them walking faster in low, declining light conditions at sunset to gather information more rapidly. Alternatively, if no decision is made at the entrance, no attempts to return to the nest would be expected, and departures may be initiated with minimal delay across all light levels and body sizes.

## 2. Methods

### (a) Experimental set-up

The experiment was conducted inside a set-up with two netted flight arenas each connected by tunnels to the centralized colony, together situated next to a 4 m tall standard-glass window for natural progression in light intensities and with UV present (electronic supplementary material, figure S1; see Supplementary materials for details). We placed a semicircular platform (22 cm diameter), displaying a red-white randomized-square pattern, horizontally at the entranceway to the connecting tunnel in each arena. The floors of the arenas were covered with ‘deadleaf’ patterning [27]. One arena had two filled sucrose feeders where bees were allowed to forage for several days prior to the experiment, motivating further foraging activity. The experimental arena had two empty feeders, for visual resemblance, and bees could enter the tunnel towards this arena but not the arena itself outside of trials. We supplemented the colony with 10 g of dry bee-collected pollen daily. Room temperature (21–23°C) and humidity (around 40%) were controlled.

We consecutively used three queen-right colonies of commercial buff-tailed bumblebees *Bombus terrestris audax* (Koppert Biological Systems, Suffolk, UK). We tested 228 individuals, with 40 repeat-measure individuals, resulting in 282 trials on 21 days. All tested bees were female foragers. We conducted trials within an hour either side of sunrise and sunset. Astronomical times for Exeter, UK were taken from www.timeanddate.com. Bees that voluntarily approached the experimental arena entranceway were allowed to emerge individually onto the platform. We recorded the light level on emergence with a lux meter (CA1110, Chauvin Arnoux, Dewsbury, UK, ± 0.1 lx), and video-recorded the trials. A trial ended when a bee that had departed the platform by flying, landed on a surface in the arena other than the platform, or after a bee attempted to return to the nest, i.e. re-enter the tunnel, three times as this suggested strong motivation to return to the nest (see electronic supplementary materials for details). We tagged each bee and subsequently measured the thorax width (inter-tegular distance) [28,29] of 184 bees (electronic supplementary material, figure S2).

### (b) Behaviours

We coded behaviours using BORIS v. 7.9.8 [30] and measured total durations per trial of frequent behaviours on the platform (walking, sitting, grooming, flight) in seconds, and the method of leaving the platform (departed or returned). We calculated the latency from emergence to departure for bees that flew away from the platform, which included the durations of walking, sitting and, rarely, grooming. To extract walking distance (*n* = 282), we developed a Python module which calculated the bee's location and classified locomotion based on movement speed (see electronic supplementary materials). We calculated average walking speed per trial by dividing this distance by the time spent walking.

### (c) Statistical analysis

We conducted all statistical analyses in R v. 4.0.1 [31,32], using the *lme4* [33] and *DHARMa* packages [34]. Generalized linear model (GLM) and linear model (LM) outputs are reported with residual degrees of freedom. In each model, light level (lux) was log-transformed to account for nonlinear responses to changes in light intensity.

We investigated the likelihood of a bee attempting to return to the nest rather than flying away under varying illumination using a binomial GLM (cloglog link), including trials ending in a return or a flight (*n* = 277). Time period (sunset and sunrise) was not considered as a fixed factor due to imbalanced sample sizes. Instead, a second similar model was fitted using only the sunset trials (*n* = 173). Finally, we tested the effects of body size and light level on the likelihood of a bee returning, with no interaction term to improve model fit (*n* = 231).

We modelled the effect of a three-way interaction between body size, time period, and light level on the time it took for bees to fly away from the platform (departure latency). We fitted two gamma-structured GLMs (inverse link): the first included measured bees that departed by flying (*n* = 217), the second included only the first recorded trial of those bees (*n* = 172). Four departure latencies of 0 s were included by adding a negligible 0.001 s to their value.

To analyse whether light level and time period interact to affect walking speed, we used a linear model (Gaussian) with the first trial of bees that both walked on the platform and departed by flying (*n* = 184 trials).

## 3. Results

Out of a total of 282 trials, 256 bees departed the platform by flying, 21 attempted to return to the colony (four at sunrise, 17 at sunset) and 198 bees (78 at sunrise, 120 at sunset) conducted only a single bout of walking and no other behaviours before departing. We observed inactivity on the platform in 41 trials, and grooming was extremely rare. Body size distributions were similar between sunrise- and sunset-emerging bees (electronic supplementary material, figure S2).

**Figure 1.**
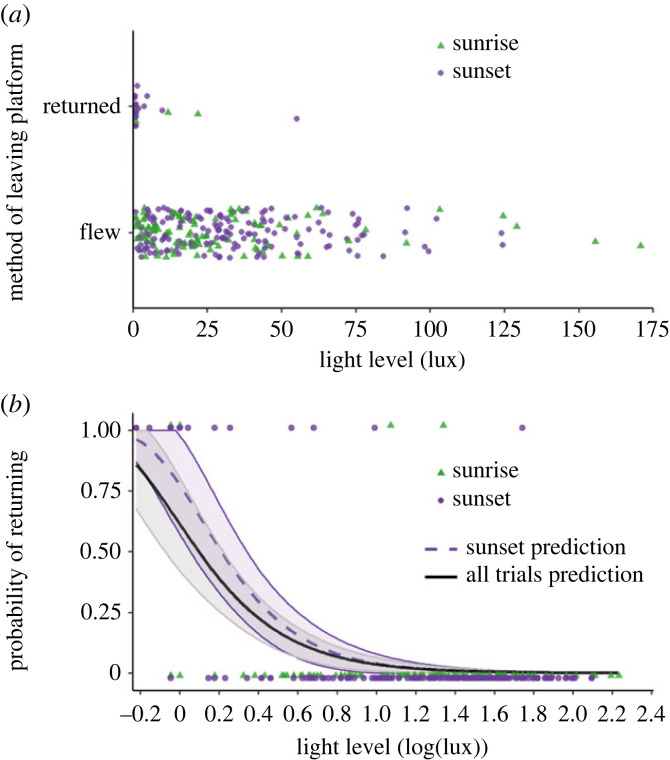
The effect of light level on the probability of a bee attempting to return to the colony. (*a*) Raw data; (*b*) log-transformed data with model predictions. Points show light levels for trials (sunrise: green triangles; sunset: purple circles, *n* = 277) where the bee departed (*y* = 0) or attempted to return to the colony (*y* = 1). Lines show model predictions for all trials (solid black) and sunset only (dashed purple) with confidence interval ribbons (1.96 × standard error).

Bees were more likely to return to the tunnel rather than fly off the platform when in lower light (under approximately 25 lx) compared to brighter light (GLM: *z*_275_ = −6.66, *p <* 0.001; figure 1). This effect was similar when considering only the sunset trials (*z*_171_ = −5.86, *p <* 0.001). Body size did not affect the likelihood of returning (*z*_228_ = −1.42, *p* = 0.155).

**Figure 2.**
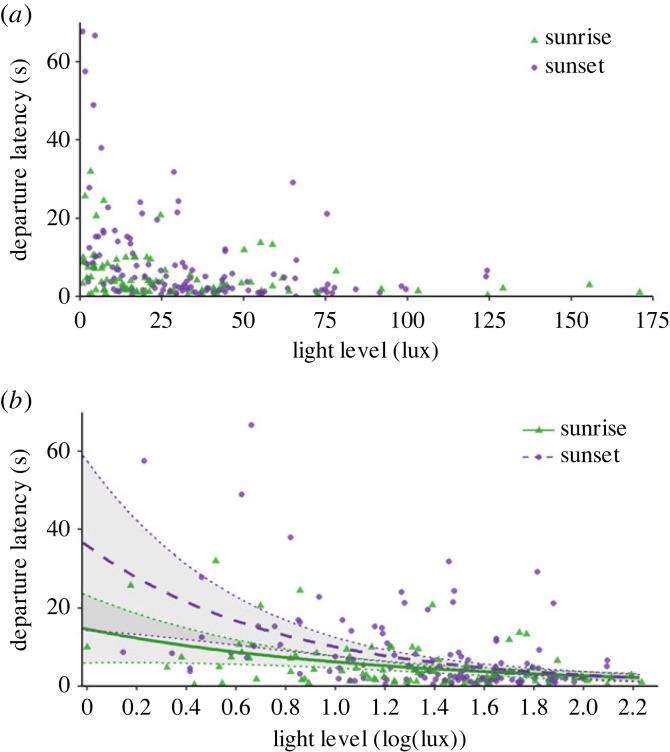
Latency to departure by flight. (*a*) Raw data; (*b*) log-transformed data with model predictions. Points show trials (sunrise: green triangles *n* = 90; sunset: purple circles *n* = 127) for size-measured bees including repeat measures. Lines show model predictions for a bee of mean thorax size (5.1 mm) for sunrise (solid green) and sunset (dashed purple) with confidence interval ribbons (1.96 × standard error).

There was a significant effect of light level on how long bees took to depart (GLM: light level: *t*_209_ = −2.15, *p* = 0.033; figure 2), but no significant effects of body size or time period, either alone or in interactions with one another (three-way interaction: *t*_209_ = −1.58, *p* = 0.117). This was also true when only one trial was considered per bee (light level: *t*_163_ = −2.12, *p* = 0.035; three-way interaction: *t*_163_ = −1.49, *p* = 0.138). Bees in lower light conditions spent longer on the platform before departing.

Bees had a mean ± standard error walking speed of 19.9 ± 0.56 mm s^−1^ (*n* = 184; electronic supplementary material, figure S4) but walking speed did not vary in relation to light, time period or their interaction (LM: interaction: *t*_180_ = −1.28, *p* = 0.201).

## 4. Discussion

Our results support the notion that bumblebee foragers assess the conditions of the external environment around sunrise and sunset. There was not a specific threshold of light under which all bees chose to return to the nest, and some flew away even under very low light. Even though the observed proportion of bees that attempted to return to the nest after accessing the platform was relatively small, the fact that they did so is indicative that ambient light conditions may play a role, and possibly together with other external factors contributed to a bee's departure decisions during sunrise and sunset. It is likely these assessments supplement information already gained before emergence from the nest under the influence of internal circadian rhythms and within-colony motivating factors. We conclude that foragers make a final decision to depart or return to the nest once they are exposed to the external environmental conditions, including ambient light.

Bees of all sizes were more likely to return to the nest in lower light levels at sunset, while returns were rare at sunrise. Generally, we expect that bees experiencing low-light conditions obtain less visual information about their environment, and may find it more difficult to navigate when foraging. Indeed, the probability of returning increased under 25 lx, which is close to their sensitivity threshold for controlled flight of 3.4 lx [26]. The differences implied between sunrise and sunset could arise from several other factors. First, bees might have less need to forage in the evening, when daytime foraging has led to colony-store replenishment. Additionally, the bee may integrate information about the time of day [35] to determine suitability of the immediate future conditions. Declining light at sunset increases risks such as being unable to fly back to the colony at the end of the bout [4], whereas the visual conditions at sunrise are improving as time passes. It may be possible for bees to assess the direction of incremental light changes; however our bees spent very short periods on the platform so may not have had time to detect these reliably. This combination of factors may result in bees making differing decisions at sunrise and sunset, even under similar light levels.

Furthermore, bees tended to be slower to depart under dimmer light and faster to depart under brighter light. Longer delays until departure under dimmer light could imply bees were taking more time to gather visual information from the environment, although the lack of effect of light level on walking speed suggests this increased latency is not compensation for increased receptor-response times or for neural integration under low light [26,36]. Contrary to expectation, we found no difference in departure latencies between sunrise and sunset or for different sized bees across light levels. It was interesting to observe that the lower sensitivity thresholds of larger bees [37] did not lead to a difference in behaviours between smaller and larger individuals, which could indicate the role of other co-variate factors at sunrise and sunset. To study this further, it would be important to know more exactly how sensitivity varies with eye and body size in bumblebees close to absolute thresholds of visual sensitivity. Notwithstanding, this result supports our prediction that departure latency is constrained by low light levels, and that the external environment is actively assessed by foragers once outside.

We conclude that assessment of the external environment is likely to be an important factor contributing to decision-making for diurnal insects experiencing changing light conditions at either end of the day. Given the importance of light intensity in the visual ecology of bees [1,4], it seems to be the most likely candidate for driving these effects. Nevertheless, in our study which aimed to observe bees under near-natural light conditions, we cannot fully rule out that close covariates of light intensity in these periods, including spectral composition and specific time of day, could also have contributed to the observed behaviours. However, it is known that insects, as shown in bees for example, have colour constancy, which makes it unlikely that they use small temporal changes in the spectrum of ambient lighting to solve visual tasks at the nest entranceway [38–40]. These changes can be more relevant when using sky compass cues to navigate [41]. It is also known that time of day is important with regard to entrained circadian rhythms [16–19] which affect activity prior to emergence from the nest and may also influence departure behaviours once outside.

Dissecting the intricate details of bee behaviour as they exit the nest provides interesting and generalizable information on the mechanisms of decision-making under variable light conditions in diurnal insects. It is also currently urgent to understand the natural behaviours of animals active around sunrise and sunset as light pollution increasingly encroaches on these times [42].

## Data Availability

The data are provided in the electronic supplementary material [43].
